# Preventing Mortality in COVID-19 Patients: Which Cytokine to Target in a Raging Storm?

**DOI:** 10.3389/fcell.2020.00677

**Published:** 2020-07-17

**Authors:** Ligong Lu, Hui Zhang, Meixiao Zhan, Jun Jiang, Hua Yin, Danielle J. Dauphars, Shi-You Li, Yong Li, You-Wen He

**Affiliations:** ^1^Zhuhai Interventional Medical Center, Zhuhai Precision Medical Center, Zhuhai People’s Hospital, Zhuhai Hospital Affiliated with Jinan University, Zhuhai, China; ^2^First Affiliated Hospital, China Medical University, Shenyang, China; ^3^Tricision Biotherapeutic Inc., Zhuhai, China; ^4^Department of Immunology, Duke University Medical University Medical Center, Durham, NC, United States

**Keywords:** COVID-19, mortality, cytokine storm, chemokine, inflammation, immunopathology, IL-6, IL-1

## Abstract

Coronavirus disease 2019 (COVID-19) from severe acute respiratory syndrome coronavirus 2 (SARS-CoV-2) infection has resulted in tremendous morbidity and mortality worldwide. A major underlying cause of COVID-19 mortality is a hyperinflammatory cytokine storm in severe/critically ill patients. Although many clinical trials are testing the efficacy of targeting inflammatory cytokines/chemokines in COVID-19 patients, the critical inflammatory mediator initiating COVID-19 patient death is undefined. Here we suggest that the immunopathological pathway leading to COVID-19 mortality can be divided into three stages with distinct clinical features that can be used to guide therapeutic strategies. Our interpretation of the recently published clinical trials from COVID-19 patients suggests that the clinical efficacy in preventing COVID-19 mortality using IL-1 blockade is subjected to notable caveats, while that for IL-6 blockade is suboptimal. We discuss critical factors in determining appropriate inflammatory cytokine/chemokine targets, timing, and combination of treatments to prevent COVID-19 mortality.

## Introduction

The current pandemic caused by newly emerged severe acute respiratory syndrome coronavirus 2 (SARS-CoV-2) has led to ∼10.8 million confirmed coronavirus disease 2019 (COVID-19) cases and more than 518,000 deaths worldwide as of July 2, 2020 (Johns Hopkins University, 2020). Severe and critically ill COVID-19 patients often demonstrate multiorgan damage including acute respiratory distress syndrome (ARDS), cardiac injury, coagulopathy, neurological impairment, gastrointestinal tract and kidney dysfunction, and have high mortality (Chen N. et al., 2020; Robba et al., 2020; Yang X. et al., 2020; Zhou F. et al., 2020). The high death rate amongst these patients is associated with SARS-CoV-2 infection-induced hyperinflammation of the innate and adaptive immune systems and the resulting cytokine storm, a cytokine release syndrome (CRS)-like syndrome, in severe cases (Channappanavar and Perlman, 2017; Cummings et al., 2020; Henderson et al., 2020; Moore and June, 2020). Cytokine storm is characterized as a rapid and prolonged systemic elevation of large quantities of inflammatory cytokines such as interleukin (IL)-6, IL-8, tumor-necrosis factor (TNF)-α, interferon (IFN)-γ, and chemokines. Accordingly, many clinical trials have been initiated to test the efficacy of neutralizing inflammatory cytokines and blocking inflammation in preventing COVID-19 mortality (Merad and Martin, 2020). Although preliminary results from some of these clinical trials are emerging, essential clinical questions remain unanswered. For example, what is the immunopathological pathway leading to COVID-19 death? What constitutes an effective strategy to target the inflammatory mediators of a cytokine storm to prevent death from COVID-19? Based on recently published data, we provide a discussion of the immunopathological pathway leading to COVID-19 mortality and the caveats of recent clinical trial results. We further suggest strategies to target the immunopathological pathway to prevent COVID-19 patient death.

## Undefined Identity of the Critical Inflammatory Mediator in COVID-19 Patient Death

The mortality of COVID-19 patients is associated with many factors. Chief among them are age, sex, and comorbidities such as hypertension, diabetes, cardiovascular disease, chronic obstructive pulmonary disease, and obesity (Chen T. et al., 2020; Cummings et al., 2020; Hajifathalian et al., 2020; Shi et al., 2020; Simonnet et al., 2020; Tian W. et al., 2020; Wu et al., 2020; Zhou F. et al., 2020). Immune parameters that are closely linked to COVID-19 mortality, established by analysis of large numbers of deceased patients, include elevated levels of IL-6, D-dimer, c-reactive protein (CRP), serum ferritin, and lactate dehydrogenase (LDH), as well as decreased lymphocyte counts and hypoalbuminemia (Cummings et al., 2020; Huang J. et al., 2020; Huang W. et al., 2020; Ruan et al., 2020; Tian W. et al., 2020; Wu et al., 2020; Yan et al., 2020; Zhou F. et al., 2020). These parameters are clearly indicative of systemic hyperinflammation and immune dysfunction in moribund COVID-19 patients.

A major unanswered question regarding the role of cytokine storm in COVID-19 fatalities is which cytokine(s) plays a critical role in the initiation of severe COVID-19. Establishing the identity of the cytokine(s) is essential for effective interventions to prevent COVID-19 patient death. Hyperinflammation caused by SARS-CoV-2 infection is similar to CRS-like syndromes in patients infected by severe influenza, SARS-CoV, and middle east respiratory syndrome-coronavirus (MERS-CoV), as well as CRS in leukemia patients receiving chimeric antigen receptor T (CAR-T) cell therapy (Channappanavar and Perlman, 2017; Moore and June, 2020). A large array of inflammatory mediators are elevated in severe/critically ill COVID-19 patients during the cytokine storm. These cytokines/chemokines are likely produced by airway epithelial cells as well as a wide array of immune cells such as macrophages, neutrophils, dendritic cells, and NK cells (Channappanavar and Perlman, 2017). These inflammatory mediators are from different molecular families, consisting of IL-1β, IL-1Rα, IL-2, sIL-2Rα, IL-4, IL-5, IL-6, IL-7, IL-8, IL-9, IL-10, IL-17, IFN-γ, TNF-α, C-X-C motif chemokine 10/interferon-gamma-induced protein 10 (CXCL10/IP10), chemokine ligand 2/monocyte chemoattractant protein-1 (CCL2/MCP-1), CCL3/macrophage inflammatory protein 1α (MIP-1α), CCL4/MIP1β, CCL5 (RANTES), granulocyte-colony stimulating factor (G-CSF), granulocyte-macrophage colony stimulating factor (GM-CSF), FGF basic, and VEGF (Chen G. et al., 2020; Gong et al., 2020; Huang C. et al., 2020; Wang F. et al., 2020; Zhao et al., 2020). The broad array of elevated inflammatory mediators during cytokine storm poses a tremendous challenge for effective intervention in COVID-19 patients. The fatal outcome of COVID-19 may be primarily due to a single cytokine (i.e., IL-6). Alternatively, several inflammatory mediators together may cause multiorgan failure and secondary hemophagocytic lymphohistiocytosis (sHLH) either concomitantly or sequentially.

Among the more than 20 inflammatory mediators elevated in severe/critically ill COVID-19 patients, IL-6 is a prime target for intervention. The role of IL-6 in CRS is supported by past clinical experience in the treatment of CAR-T therapy-induced cytokine storm (Moore and June, 2020). Moreover, elevated IL-6 levels are associated with COVID-19 severity and mortality (Cummings et al., 2020; Han et al., 2020; Ruan et al., 2020; Tian W. et al., 2020). However, clinical data also indicates that the plasma levels of IP-10 and MCP-3, but not IL-6, are strongly correlated with disease severity and fatal outcomes (Yang Y. et al., 2020). Interestingly, plasma IL-6 levels were either not different (Huang C. et al., 2020), or only slightly different in the early phase of disease progression (Yang Y. et al., 2020) between mild and severe/critically ill COVID-19 patients. Importantly, elevation of IL-6 levels in severe COVID-19 patients was later than that of CCL5 (Han et al., 2020). Furthermore, in a daily transcriptomic profiling of whole blood from COVID-19 patients, the mRNA expression levels for most of the examined inflammatory genes including IL-6, except the IL-1 family, which are elevated early, reached peaks after respiratory function nadir (Ong et al., 2020). These clinical data raise the critical question of which member(s) of the COVID-19 cytokine storm serves as an initiator of the deadly immunopathological process.

## Three Stages of Immunopathological Pathway to COVID-19 Mortality: Initiation, Amplification, and Consummation

Mortality in COVID-19 patients results from fatal pneumonia and damage to other vital organs. Pathological studies of deceased COVID-19 patients revealed edematous lungs with necrotic lesions. The lung tissues exhibited diffuse alveolar damage with formation of hyaline membranes, thrombosis and microangiopathy in the small vessels and capillaries, wide intra-alveolar hemorrhage, vascular congestion, and infiltration of inflammatory cells including inflammatory monocytes and CD4^+^ and CD8^+^ T lymphocytes (Adachi et al., 2020; Barton et al., 2020; Early et al., 2020; Fox et al., 2020; Martines et al., 2020; Wang C. et al., 2020; Xu Z. et al., 2020). Extensive neutrophil infiltration was also observed in lung tissue of some deceased patients (Barnes et al., 2020; Fox et al., 2020; Tian S. et al., 2020; Xu Z. et al., 2020). Furthermore, myocyte necrosis was observed in the hearts (Fox et al., 2020), and lobular lymphocyte infiltration with patchy necrosis was seen in the livers of the patients (Tian S. et al., 2020). These pathological findings are consistent with clinical observations of multiorgan dysfunction in severe/critically ill COVID-19 patients (Chen N. et al., 2020; Yang X. et al., 2020; Zhou F. et al., 2020) and suggest a similar pathological process of hyperinflammation to those seen in fatal influenza and SARS infections (Nicholls et al., 2003; Kuiken and Taubenberger, 2008; Gill et al., 2010; Mauad et al., 2010).

**FIGURE 1:**
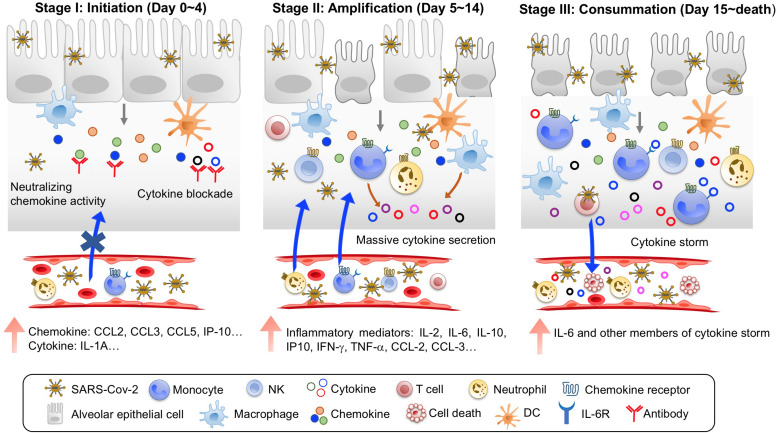
Three stages of immunopathological pathway leading to COVID-19 mortality. Stage I is Initiation, with an early induction of predominant chemokines upon SARS-CoV-2 infection and viral sepsis. Treatment at this stage with blocking agents to chemokines and emerging inflammatory cytokines is the key to prevent COVID-19 mortality. Stage II is Amplification, with large production of many more inflammatory mediators and massive recruitment of inflammatory cells to amplify immunopathological process. Stage III is Consummation, with continuous increases of inflammatory mediators and widespread organ damages.

Although pathological and clinical findings from deceased COVID-19 patients imply a critical role for the hyperinflammatory cytokine storm in their mortality, the immunopathological pathway leading to patient death remains undefined. Based on recent clinical studies and multi-omics analysis, we propose that the immunopathological pathway leading to COVID-19 mortality consists of three stages, with stage 1 as the initiation phase, stage 2 as the amplification phase, and stage 3 as the consummation phase (Figure 1). These three stages progress rapidly, with a median time from onset of disease to death of 16–18.5 days (Chen T. et al., 2020; Yang X. et al., 2020; Zhou F. et al., 2020), and progression is further accelerated by comorbidities such as cardiovascular disease (Shi et al., 2020).

**Stage 1:** The initiation phase occurs in the first 4 days of disease for fatal COVID-19 cases. During this phase, several major features can be identified. First, clinical symptoms are not severe, but SARS-CoV-2 replicates swiftly, causing viremia in patient blood (Chen W. et al., 2020; Chen X. et al., 2020; Li et al., 2020). Second, compared to COVID-19 survivors, non-survivors demonstrate early signs of systemic inflammation, with significantly elevated LDH and serum ferritin and decreased peripheral lymphocyte counts (Zhou F. et al., 2020). Interestingly, serum IL-6 levels in non-survivors were not significantly different from those in survivors at this stage (Zhou F. et al., 2020), suggesting that IL-6 may not be among the first inflammatory mediators. Consistent with this notion, plasma levels of CCL-2 and IP-10 were elevated earlier than IL-6 in intensive care unit (ICU) COVID-19 patients (Young et al., 2020). Furthermore, peripheral blood levels of CCL5 and IL-10 were increased earlier than IL-6 and IFN-γ in severe COVID-19 patients (Zhao et al., 2020). Third, the initial group of inflammatory mediators are likely dominated by chemokines in moribund patients at this stage. In a longitudinal study on SARS-CoV-2 infection of ferrets, upper respiratory cell populations first upregulated CCL8 and CCL9, and cells from nasal wash had increased expression of CCL2, CCL8, CCL9, and CCR5 but not IL-6 at day 3 post-infection (Blanco-Melo et al., 2020). The peripheral blood levels of CCL5 were elevated before IL-6 in severe COVID-19 patients (Zhao et al., 2020). Furthermore, several multi-omics analyses of bronchoalveolar lavage fluid (BALF) from COVID-19 patients revealed chemokine-rich signatures including the expression of CCL2, CCL3, CCL4, CCL7, CCL8, CXCL2, CXCL8, CXCL17, and IP-10 (Bost et al., 2020; Xiong et al., 2020; Zhou Z. et al., 2020). Thus, the immunopathological process during stage 1 of fatal COVID-19 is likely initiated by rapid replication of SARS-CoV-2 and the predominant induction of chemokines in lung tissues. The infection-induced chemokines then begin to recruit innate and adaptive immune cells to lung lesions (Figure 1).

**Stage 2:** The amplification phase occurs at day 5–14 after disease onset in fatal COVID-19 cases. During this period, disease features are characterized by amplification of inflammatory immune responses and quick progression to severe/critical illness. First, patients display severe symptoms, including dyspnea (5–7 days after disease onset), sepsis (10 days after disease onset), and ARDS (8–12 days after disease onset; Li et al., 2020; Wang D. et al., 2020; Zhou F. et al., 2020). Admission to ICU is needed to maintain respiratory function. Second, there is a massive recruitment of inflammatory cells including macrophages, neutrophils, DCs and lymphocytes to the lung. The predominant production of chemokines during the initiation phase serves to recruit inflammatory innate and adaptive immune cells including neutrophils, inflammatory monocytes, and pathogenic T lymphocytes to the lung lesions. Consistent with postmortem pathology (Barnes et al., 2020; Fox et al., 2020; Tian S. et al., 2020; Xu Z. et al., 2020), analyses of BALF from COVID-19 patients revealed lung infiltration of multiple immune cell types, including neutrophils, M1 macrophages, activated mast cells/NK cells/DCs, CD4^+^, and CD8^+^ T lymphocytes (Bost et al., 2020; Zhou Z. et al., 2020). Importantly, BALF from severe COVID-19 patients was highly enriched for neutrophils, FCN1^+^ monocytes, and inflammatory monocyte-derived macrophages (Bost et al., 2020; Liao et al., 2020). Third, inflammatory mediators surge systemically. Multiple studies have shown that the peripheral levels of inflammatory mediators including IL-2, IL-2Rα, IL-6, IL-7, IL-10, GSCF, IP10, TNF-α, CCL-2, CCL-3, CRP, and D-dimer were dramatically enhanced in critically ill and deceased patients (Chen G. et al., 2020; Huang C. et al., 2020; Zhou F. et al., 2020). Systemic inflammatory mediators could come from the lung or peripheral cells (Wilk et al., 2020; Zhou Y. et al., 2020). Therefore, the immunopathological process during stage 2 of fatal COVID-19 is characterized by rapid deterioration of lung function, likely caused by large-scale inflammatory infiltration-induced damage (Figure 1).

**Stage 3**: The consummation phase occurs from day 15 until death, with a total median time of 16–18.5 days from onset to fatality in severe/critically ill COVID-19 patients (Chen T. et al., 2020; Yang X. et al., 2020; Zhou F. et al., 2020). A hallmark for patients entering this phase is the appearance of severe damage in other organs including heart, kidney, and liver in addition to ARDS (Yang X. et al., 2020; Zhou F. et al., 2020). All patients at this stage require invasive mechanical ventilation for life support. Peripheral neutrophil counts further increase, while lymphopenia worsens. Importantly, continued rise of systemic IL-6 and other members of the cytokine storm in non-survivors (Wang D. et al., 2020; Yang X. et al., 2020; Zhou F. et al., 2020) contributes to vascular leakage, complement cascade activation, and overt disseminated intravascular coagulation (DIC) that was found in 71.4% of non-survivors but only in 0.6% of survivors (Tang et al., 2020). Patients die by multiorgan failure. In summary, the inflammatory mediators of the cytokine storm in moribund COVID-19 patients may play coordinated roles in the immunopathological pathway leading to death. Accurate identification of the roles of the individual members of the cytokine storm is needed to develop effective interventions.

## The Efficacy of Inflammatory Cytokine Blockade in Preventing COVID-19 Mortality: Caveat and Suboptimality

Multiple clinical trials targeting inflammatory mediators including IL-1, IL-6, TNF-α, GM-CSF, M-CSF, IFN-γ, JAK1/JAK3, CCR2, CCR5, and complement C3/C5 are underway to treat COVID-19 patients [summarized in reference (Merad and Martin, 2020)]. Preliminary reports with retrospective and prospective cohorts describe the effects of blockade of IL-1, GM-CSFRα and IL-6 in COVID-19 patients. Two studies using the IL-1 receptor antagonist anakinra to treat COVID-19 reported reduced mortality rates (Cavalli et al., 2020; Huet et al., 2020). In a retrospective cohort study, 29 patients receiving high-dose anakinra plus standard treatment had a mortality of 10% (3/29) at 21 days after initiation of treatment while 16 control patients receiving only standard treatment had a mortality of 44% (7/16; Cavalli et al., 2020). In a second study, consisting of a prospective cohort for anakinra treatment and a retrospective control group, the rate of death or admission to ICU for invasive mechanical ventilation was 25% (13/52) for the anakinra treatment cohort but 73% (32/44) for historical control group (Huet et al., 2020). These preliminary results are encouraging but have some notable caveats. First, these were small-scale studies, and the risk factors associated with COVID-19 mortality in both studies were unfavorably biased toward the control groups. In the first study, median ferritin levels were 2218 ng/ml for the control group while 1237 ng/ml for the anakinra treated group (Cavalli et al., 2020). For comparison, two groups of 113 and 54 deceased COVID-19 patients had median ferritin levels of 1418.3 and 1435.3 ng/ml, respectively (Chen T. et al., 2020; Zhou F. et al., 2020). In the second study, the body-mass index (BMI) was 29.0 for the control historical group and 25.5 for the anakinra-treated group. Obesity is an independent predictor of respiratory distress requiring invasive mechanical ventilation or death from COVID-19 (Hajifathalian et al., 2020; Simonnet et al., 2020). Second, both studies combined anakinra with hydroxychloroquine to treat the experimental groups. Although a clinical benefit of hydroxychloroquine has yet to be established and is currently being tested in large scale clinical trials, the potential treatment efficacies from both studies could have derived from a combinatorial effect of anakinra plus hydroxychloroquine. Thus, further randomized controlled trials are needed to address these caveats.

A recent prospective study from a single center reported the outcome of GM-CSF blockade in severe COVID-19 patients with anti-CSFRα antibody mavrilimumab (De Luca et al., 2020). Of 13 non-mechanically ventilated patients treated with mavrilimumab plus standard treatment, no patient died. In contrast, 7 of 26 patients receiving standard treatment died during a 28-day period, suggesting that GM-CSF blockade may reduce cytokine storm induced mortality. Large-scale controlled studies are needed to overcome deficiencies of the small sample size, lack of randomization and a short follow-up time.

Many reports have presented the clinical efficacy of IL-6 blockade in treating ∼1600 severe/critically ill COVID-19 patients using the anti-IL-6R monoclonal antibodies tocilizumab (Alattar et al., 2020; Capra et al., 2020; Guaraldi et al., 2020; Ip et al., 2020; Klopfenstein et al., 2020; Knorr et al., 2020; Morena et al., 2020; Perrone et al., 2020; Rojas-Marte et al., 2020; Toniati et al., 2020; Xu X. et al., 2020), or sarilumab (Benucci et al., 2020), and IL-6 neutralizing antibody siltuximab (Gritti et al., 2020). These clinical studies have demonstrated the following effects in the large population of COVID-19 patients: 1. A significant fraction of treated patients have improved clinical symptoms such as fever, respiratory function, and corresponding lung imaging. 2. Inflammation markers such as neutrophil counts and CRP are significantly reduced, and lymphocytes are increased. 3. Mortality rates and admission to the ICU are reduced in studies specifically designed to test these outcomes (Guaraldi et al., 2020; Ip et al., 2020; Perrone et al., 2020). The clinical results are very encouraging given the severe and critical clinical conditions of the treated patients. However, several important points are worth noting. First, the clinical efficacy of blocking IL-6 signaling in preventing mortality of COVID-19 patients is suboptimal. In two large scale studies, including a multi-center phase II trial using death rate as the primary outcome, mortality rates were reduced by 10% (56% to 46%; Ip et al., 2020), and 15% (35% to 20%; Perrone et al., 2020) at a cutoff of 30-days, indicating a majority of the moribund patients [82% (Ip et al., 2020) and 57% (Perrone et al., 2020)] still succumbed. Second, all patients received concomitant antiviral treatment including hydroxychloroquine, remdesivir, lopinavir-ritonavir, ribavirin, and/or IFN-α2A as well as anti-bacterial/fungal drugs. Some patients also received anti-inflammatory glucocorticoids. Thus, the clinical efficacy in the patients with improved outcomes likely derives from a combination of these treatments.

In summary, the interpretation of the clinical efficacy of IL-1 and GM-CSF blockade is subject to major caveats, while the clinical efficacy of IL-6 blockade is suboptimal.

## Strategies for Targeting Cytokine Storm to Prevent COVID-19 Mortality

The caveats and suboptimal outcomes associated with the clinical results from IL-1 and IL-6 blockade in preventing COVID-19 mortality raise important questions regarding the optimal target(s) and strategies to treat a cytokine storm. As outlined above in the three stages of the immunopathological pathway leading to COVID-19 mortality, inflammatory mediators serve different roles in different phases. Choosing the correct target at the right time, combined with the right supplemental treatments is essential to preventing COVID-19 death.

### Choice of Targets

For the more than 20 elevated inflammatory cytokines/chemokines in the cytokine storm in COVID-19 patients, clinical trials are underway to test the clinical efficacy of blocking at least 10 of them (Merad and Martin, 2020). Given the predominant chemokine signature in the initiation phase of COVID-19 immunopathology, neutralizing chemokine activity will reduce the massive pulmonary recruitment of inflammatory monocytes/macrophages/neutrophils and prevent mortality. It was previously shown that CCL2 recruits CCR2^+^ inflammatory monocytes to the lung during severe influenza infection and prophylactic use of CCR2 antagonist reduces pulmonary immunopathology, markedly improving the survival of influenza infected mice (Lin et al., 2008, 2011). Similarly, antibody-mediated blocking of CCL3 (MIP-1α) dramatically reduced lung pathology and mortality in mice lethally infected with pneumovirus, a mouse mimic of human respiratory syncytial virus (Bonville et al., 2003). Thus, inflammatory initiators of lethality in viral respiratory infections are excellent targets and should be prioritized in trials to prevent COVID-19 mortality. The current data suggest that chemokines such as CCL2, CCL3, CCL5, and IP10 are the initiators of the deadly COVID-19 immunopathological pathway (Figure 1).

### Choice of Timing

Sufficient evidence supports the notion that timing is of the essence in targeting inflammatory mediators during COVID-19 cytokine storm. We define the initiation phase of the lethal immunopathology in fatal COVID-19 patients as the first 4 days after disease onset, when IL-6 levels first begin to increase, and the amplification phase can be avoided by timely interventions (Zhou F. et al., 2020). In lethal infections of mice with pneumovirus, surprisingly, ribavirin treatment inhibited pneumovirus replication but failed to reduce lung inflammation and mortality, suggesting early intervention is essential to stop the deadly pulmonary immunopathology program. Consistent with this notion, several small clinical studies using IL-6R inhibitors to treat COVID-19 patients at different stages showed opposite outcomes. When 8 patients were treated with sarilumab 24 h after hospitalization, 7 of them were discharged within 14 days (Benucci et al., 2020). In contrast, patients having CRS treated with tocilizumab worsened into sHLH and died (Radbel et al., 2020). Furthermore, limited clinical improvement was observed when tocilizumab was used in severe COVID-19 patients (Knorr et al., 2020) and mortality was not significantly lowered in severe to critically ill patients (Rojas-Marte et al., 2020). As the ∼1600 COVID-19 patients received IL-6/IL-6R blocking mAbs at the severe and critical stages, it is reasonable to expect that early treatment at stage 1 would have better outcomes. Thus, it is strongly suggested that the current treatment guideline be modified to begin treatment very early in COVID-19 patients (Figure 1). This may be especially important for high-risk patients (Chen T. et al., 2020; Cummings et al., 2020; Hajifathalian et al., 2020; Shi et al., 2020; Simonnet et al., 2020; Tian W. et al., 2020; Wu et al., 2020; Zhou F. et al., 2020).

### Choice of Combination

Combining two or more agents targeted against the elevated inflammatory mediators of COVID-19 may enhance their efficacy in preventing mortality. The suboptimal efficacy of IL-6 blockade in reducing mortality further indicates an urgent need to develop combination therapies. However, there is almost a complete lack of scientific understanding of the individual and synergistic roles of these inflammatory cytokines/chemokines in COVID-19 mortality. Although combination therapy of IL-1 and IL-6 blockade is already in clinical trial (Merad and Martin, 2020), the dynamic expression pattern of these two cytokines has not been clearly defined (Ong et al., 2020). Thus, a pertinent question regarding their combination therapy is how to use the IL-1 and IL-6 blocking agents, concomitantly, or sequentially?

The choice of combinations of anti-inflammatory and antiviral agents should also be carefully considered. Severe SARS-CoV-2 infection induces a low IFN-I and III response (Blanco-Melo et al., 2020; Bost et al., 2020; Hadjadi et al., 2020), providing a potential mechanism for the swift replication of SARS-CoV-2 and viremia in moribund COVID-19 patients. Thus, it may be helpful to apply type I and III IFNs early in high-risk COVID-19 patients to control virus replication (Lokugamage et al., 2020; Mantlo et al., 2020; Prokunina-Olsson et al., 2020). A caveat that needs consideration in using type I and III IFNs is their potential damage to lung epithelial repair as recently reported (Broggi et al., 2020; Major et al., 2020). Nevertheless, early and aggressive use of combined antiviral and anti-inflammatory agents in high-risk COVID-19 patients can halt the progression of the deadly immunopathological program and prevent mortality (Figure 1).

## Conclusion

Recent clinical and multi-omics studies on COVID-19 patients suggest a rapid onset of the immunopathological pathway leading to mortality. Early application of antiviral and anti-inflammatory agents in high-risk COVID-19 patients is essential to prevent death.

## Data Availability Statement

The original contributions presented in the study are included in the article/supplementary material, further inquiries can be directed to the corresponding author/s.

## Author Contributions

Y-WH and LL contributed to conception. Y-WH wrote the manuscript. HZ generated the figure. DD edited the manuscript. MZ, JJ, HY, S-YL, and YL participated in discussion and improvement of the perspective. All authors contributed to the article and approved the submitted version.

## Conflict of Interest

Y-WH and S-YL are shareholders of Tricision Biotherapeutic Inc. The remaining authors declare that the research was conducted in the absence of any commercial or financial relationships that could be construed as a potential conflict of interest.
